# MOTIVATIONAL FACTORS AFFECTING THE INTENTION TO VOLUNTEER IN ELDER CARE AGENCIES: A PILOT STUDY

**DOI:** 10.1093/geroni/igac059.2150

**Published:** 2022-12-20

**Authors:** Rong Fu, Qi Xu

**Affiliations:** Siena College, Loudonville, New York, United States; China Agricultural University, Beijing, Beijing, China (People's Republic)

## Abstract

The rapid rise of the aging population and the “4-2-1” family structure in China calls for speeding up the construction of the elderly care service system. To facilitate this process, it is desirable to encourage voluntary participation in elder care agencies. This study aims to identify motivational factors that may affect the intention to volunteer in elder care agencies in China. Data were collected in a sample of 199 residents of Beijing, China. Our results revealed gender differences in the demographic and socioeconomic factors that affect the volunteer intention. For men, earning more than 12,000 Yuan (about $1900) significantly increased the intention to participate in voluntary services (p = .043). For women, those younger than 60 years of age were more willing to participate in voluntary services than their older counterparts (p = .014). Participants identified three key motivational factors for providing volunteer services in elder care agencies: (1) to show love and kindness to older adults; (2) to fulfill oneself and take social responsibilities; and (3) to gain more life experience. We also found that most participants prefer to provide spiritual comfort services (e.g., chatting, shopping) and life care services (e.g., cooking, house cleaning) than other types of services. Our study suggested that effective interventions should be designed and implemented to match volunteering resources to the needs of elder care agencies in China.

